# Cases of Scarlet Fever

**Published:** 1887-05

**Authors:** H. Christopher

**Affiliations:** St. Joseph, Mo.


### CASES OF SCARLET FEVER.

BY H. CHRISTOPHER, M. D., OF ST. JOSEPH, MO.

It is unfortunate for patients that theories give rise to plans of treatment. Were they always true, this would not be so objectionable; but it is no news to state that the history of medicine discovers many grave-yards of theories. They perished and were buried because there was no life in them. Truth is immortal, but human judgments evanescent, unless they happen to conform to truth. Just now the profession is strongly inclined to the germ theory of disease, and treatment is antiseptic, in a curative sense, for everything, because germs are thought to be the primary cause of all forms and kinds of disease. How many victims will perish on account of this theory even Time will not be able to tell. It has afforded no light in the treatment of disease, because old methods are as successful now as formerly, for the very good reason that *principles* never change. What is true at one time will be always true; and, so far as the art of medicine is based on true principles, it will so continue. Changes in modes and methods do occur in the progress of the art, but they come because the old were found to violate the fundamental principles of the art. Knowledge discovers errors.

In A. D. 1885 two cases of scarlet fever occurred in my family. Their occurrence could not be traced to a previous case, or any local cause as an excitant. One was very mild. The patient was a boy of about three years. He was not confined to his bed a single day ; only to his room. The other—a girl of about seven years—had a severe attack, which preceded the other a day or two. The eruption embraced the whole surface at the same time. The skin was very much flushed, dry, and rough ; the fever high, with disturbed dreams in sleep ; the throat and nose were involved, the former highly inflamed, and the latter very dry. She had gone to bed the night before with a high fever, but there was no appearance of an eruption, nor was any eruptive disease thought of or suspected. She was given a purgative dose of calomel at bedtime. In the morning, when awakened, she was covered with the scarlet rash from face to feet ; the fever was still high. The treatment consisted of laxative doses of calomel until the fever was lessened, and anointing the whole body with vaseline, ℥i, and resorcin, grs. v, applying it every night and washing alternate nights. The throat and nose were sprayed with the same vaseline night and morning. The recovery was without sequelæ, and with but little desquamation, under the usual time—about a week. There was in the house at the same time, and in an adjoining room, a boy about thirteen years old, who was transferred to another room during the attack, and for a time after. He escaped at that time. The rooms and halls of the house were fumigated with sulphurous oxide and chlorous oxide, produced by the action of strong sulphuric acid on the potassium chlorate. This I had used on several accasions to disinfect close cellars, and with the effect of removing all detectable odors. This fumigation was done about the 1st of November.

On Christmas day, 1885, a daughter, with her two children, came on a visit. I thought that there was no risk, as the house had been ventillated every day since its fumigation. She occupied a room that had not been entered by either of the patients after they recovered ; the children, however, went into every room of the house, after they came. On the 2d of January, the younger of the children—three years, past—was found to have the fever on awaking that morning, and on the 6th the elder was attacked ; both rather mild cases. The younger soon recovered, but the elder lingered, showing that the specific poison was not yet eliminated from the body. There was a slight anasarca and enlargement of a cervical gland, and pallor of the face. The recovery was slow ; treatment on general principles. At one time the case looked threataning, ecchymotic spots appearing on the ankles, with considerable fever. A few grains of calomel were given at bedtime, and the next day ¼-grain doses of quinine every half hour. In twenty-four hours the ecchymotic spots were much fainter, the fever but slight, and the general condition much improved, so that I considered all danger passed. In these cases the throat and nose were sprayed, night and morning, with resorcin-vaseline, and their bodies anointed with the same. The recovery of the elder was complete, leaving no sequelæ about the nose, ears, throat or glands.

On the 8th of January the thirteen-year old boy had a slight flush on the left side of the neck, with a little fever. The eruption was regarded as scarlatina, and in a day or so the tongue confirmed the conclusion. The eruption did not extend, and in a few days he was regarded as well. He was not in bed a day, and his diet was changed but little. On the 15th, I was much surprised to find him with fever again, and his whole body covered with the scarlet rash, with every appearance of a fresh attack. The case was treated as the others, and in the usual time the fever and the rash disappeared. The tongue was correspondingly affected, the peculiar scarlatinous tongue being very marked.

The facts noteworthy in these cases are: 1. That the fumigation and two months' ventillation did not destroy the virus; 2. That the eruption in the case of the 13-year old boy re-appeared on the eighth day after the first appearance, and with greater violence than at first, and continued the usual time; 3. That all the cases were treated on *general principles*, and that treatment proved as effective and as rapid as any supposed specific treatment. The resorcin and vaseline were employed because the combination was known to relieve inflammatory action of the skin, as in denuded burns, and not because of any antiseptic properties they possess. I use the combination constantly in nasal catarrh, especially the atrophic form, and when the catarrh is attended by any disagreeable odor. Its excellent remedial virtues are also seen in any form or kind of ulcerative action on the skin or mucous surfaces.

